# Milk Fat Globule Membrane Attenuates Acute Colitis and Secondary Liver Injury by Improving the Mucus Barrier and Regulating the Gut Microbiota

**DOI:** 10.3389/fimmu.2022.865273

**Published:** 2022-06-21

**Authors:** Zhenhua Wu, Xiaoyi Liu, Shimeng Huang, Tiantian Li, Xiangyu Zhang, Jiaman Pang, Junying Zhao, Lijun Chen, Bing Zhang, Junjun Wang, Dandan Han

**Affiliations:** ^1^ State Key Laboratory of Animal Nutrition, College of Animal Science and Technology, China Agricultural University, Beijing, China; ^2^ Academy of National Food and Strategic Reserves Administration, Beijing, China; ^3^ National Engineering Center of Dairy for Early Life Health, Beijing Sanyuan Foods Co. Ltd., Beijing, China; ^4^ Key Laboratory of Animal Epidemiology of the Ministry of Agriculture, College of Veterinary Medicine, China Agricultural University, Beijing, China

**Keywords:** milk fat globule membrane, colitis, hepatic injury, mucus barrier, gut microbiota

## Abstract

**Objective:**

Inflammatory bowel disease (IBD) often occurs along with extraintestinal manifestations, including hepatic injury. Milk fat globule membrane (MFGM) is an active substance with a potential anti-inflammation activity. However, its alleviated effect and mechanisms in IBD as well as the IBD-induced secondary liver injury are still unclear.

**Methods:**

C57BL/6J mice were administered with a 21-day oral gavage of MFGM, followed by 7 days of drinking water with 4% dextran sulfate sodium (DSS). Disease activity index (DAI), histological features, and cytokines of the colon and liver were evaluated. Then, RNA-seq of the colon and liver was conducted. The gut microbiota was assessed by analyzing 16S rRNA gene sequences, and finally the integrity and the function of the mucus barrier were evaluated by Alcian blue staining, real-time quantitative PCR, and ELISA.

**Results:**

Prophylactic MFGM treatment was effective against colitis to include effects in body weight loss, DAI score, colonic length, intestinal pathology, and histological score. Additionally, prophylactic MFGM decreased the levels of interleukin (IL)-1β, IL-6, and myeloperoxidase in colonic tissue, while it increased the IL-10 level. Moreover, the gene expressions of *MUC2*, *MUC4*, *Reg3b*, and *Reg3g* associated with the production of the molecular mediator of immune response, membrane invagination, and response to protozoan were strikingly upregulated when administered with MFGM. On the other hand, the beneficial effects of MFGM were related to the enriched abundance of genera such as *Faccalibacumum* and *Roseburia* in feces samples. Consistently, the administration of MFGM was also found to alleviate DSS-induced hepatic injury. Furthermore, the glutathione transferase activity pathway was enriched in the liver of MFGM-treated mice after DSS administration. Mechanistically, prophylactic MFGM enhanced the mucosal barrier by increasing the gene levels of *Reg3b* and *Reg3g*. Meanwhile, the alleviation of MFGM on liver injury was dependent on the reduced hepatic oxidative stress.

**Conclusions:**

MFGM attenuated colitis and hepatic injury by maintaining the mucosal barrier and bacterial community while inhibiting oxidative stress, which might be an effective therapy of hepatic injury secondary to IBD.

## Introduction

Inflammatory bowel disease (IBD), characterized by uncontrolled immune response, diarrhea, body weight loss, and rectal bleeding, is becoming more prevalent worldwide in recent years (1–3). The development and pathogenesis of IBD is influenced by genetic, dietary, and environmental factors as well as gut microbiota (4–7). IBD patients always suffer from various extraintestinal manifestations (8, 9). Nowadays, approximately 5% of IBD patients also develop further liver disorders and various hepatobiliary diseases, including fatty liver, autoimmune hepatitis, and cirrhosis (9, 10). The intestinal homeostasis (11, 12) and extraintestinal manifestations (7, 13, 14) are intimately linked with intestinal mucosal barrier function and gut microbiota. In fact, the physiological position of the liver provides its close interaction with the gut, so that the gut–liver axis, consisting of gut microbiota, intestinal barrier, and hepatic immune, attracts attention (14, 15). The gut–liver axis is widely considered to be associated with hepatic injury (14, 16–18). The intestinal epithelia secreted antimicrobial proteins, such as the intestinal C-type regenerating islet derived-3 (Reg3) lectins, to defend against pathogens and keep commensal bacteria in the intestinal cavity (13, 19). Moreover, the deficiency of *Reg3b* and *Reg3g* contributes to bacterial translocation and liver disease (19–21). Recent studies also demonstrated that the alternation in the gut microbiota may contribute to the abnormal gut–liver axis (14, 15). However, the underlying mechanisms and therapeutic targets of colitis-associated liver injury are poorly known.

Milk fat globule membrane (MFGM), the component that surrounds fat globules in milk, has its beneficial effects on gut function, immune boosting, and cognitive development (22–25). However, it is still unclear whether and how MFGM protects from colitis and secondary liver injury. To demonstrate it, acute colitis was induced in mice along with colitis-associated liver damage by dextran sulfate sodium (DSS) after the pre-supplementation of MFGM (26, 27). We hypothesize that dietary supplementation of MFGM attenuates colitis, and colitis-associated hepatic damage is mediated though the modulation of the gut microbiota.

## Experimental Section

### Animal Experiments

**Figure 1:**
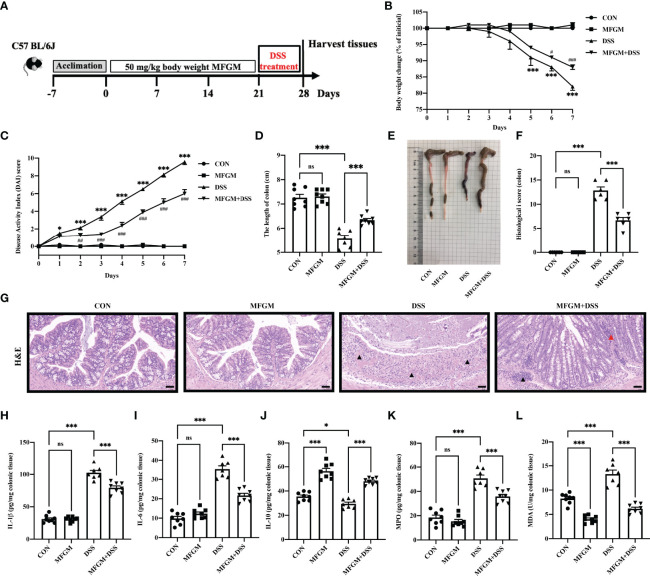
Milk fat globule membrane (MFGM) alleviated dextran sulfate sodium (DSS)-induced experimental colitis. **(A)** Diagram illustrating the experimental design employed in this study. Mice were treated with oral phosphate-buffered saline (PBS) or MFGM for 3 weeks before 4% dextran sulfate sodium in drinking water. **(B)** Daily body weight changes throughout the DSS treatment duration of the study. **(C)** Kinetics of daily disease activity index scores throughout the DSS treatment duration of the study. Data were presented as means ± SEM (*n* = 6–8 per group). Statistical significance was determined using one-way ANOVA, followed by Tukey’s test. **P ≤0.01, ***P ≤0.001 relative to control group. ^#^P ≤ 0.05, ^##^P ≤ 0.01, ^###^P ≤ 0.001 relative to DSS group. **(D)** Length of colon from each group and **(E)** macroscopic pictures of colons (*n* = 6–8 per group). **(F)** Histological scores of colons, and **(G)** H&E-stained colon sections (*n* = 6 per group). Scale bars represent 100 μm. The infiltration of immunocytes was marked by black triangles, and local bleeding was marked by red triangles. Concentrations of three representative pro-inflammatory cytokines—IL-1β **(H)**, IL-6 **(I)**, and IL-10 **(J)**—in the colon. Concentrations of myeloperoxidase **(K)** and malondialdehyde **(L)** in the colon. Data are presented as means ± SEM (*n* = 6–8 per group). Statistical significance was determined using one-way ANOVA, followed by Tukey’s test. ns, no significant, *P ≤ 0.01, ***P ≤ 0.001.

The experimental design is shown in **Figure 1A**. MFGM was obtained from Beijing Sanyuan Foods Co., Ltd. Six- to seven-week-old specific pathogen-free (SPF) C57BL/6J male mice (obtained from SPF Biotechnology Co., Ltd., Beijing, China) were maintained in a standard SPF facility with 12-h light and 12-h dark cycles at 22°C at four animals per cage. After a week of acclimation, the mice were randomly divided into four groups (*n* = 8) for the subsequent experiment: CON group [oral gavage of 200 μl sterile phosphate-buffered saline (PBS) for 4 weeks with regular tap water], MFGM group (oral gavage of 200 μl 50 mg/kg body weight MFGM in sterile PBS for 4 weeks with regular tap water), DSS group (oral gavage with 200 μl sterile PBS for 4 weeks with 4% DSS in drinking water for the last week), and MFGM + DSS group (oral gavage with 200 μl 50 mg/kg body weight MFGM in sterile PBS for 4 weeks with 4% DSS in drinking water for the last week). The dose of MFGM is based on our previous study in other animal models (25, 28). The animal experiment was performed in accordance with the guidelines of the local ethics committee. The disease activity index (DAI) score was evaluated to assess the severity of colitis by combining the scores of body weight loss, diarrhea of the stool, and the extent of blood in the feces (29). Body weight and DAI were recorded daily in the last week. After sacrifice, the length of gross colon was recorded, and 5-mm segments of the mid-colon and liver were fixed in formalin for sectioning and staining. Fecal samples, serum samples, and colonic and hepatic tissue from each mouse were collected and immediately stored at -80°C for a subsequent analysis.

### Histological Analysis, Alcian Blue Staining, and Immunofluorescence Staining

For morphological measurements, the fixed colonic and hepatic tissue were paraffin-embedded, dehydrated, sectioned at 5 μm, and stained with hematoxylin and eosin (H&E). The histological score was consisting of the extent of inflammatory infiltration, histopathological changes, ulceration and loss of crypt, and the completeness of colonic epithelia (3). To measure the thickness of mucus, the colonic sections were stained with Alcian blue for 10–15 min and dehydrated with 100% alcohol and xylene.

Immunofluorescence staining of proliferating cell nuclear antigen (PCNA) was performed on colonic sections of mice with the PC10 antibody (Abcam, ab201672). Immunofluorescence staining of F4/80 was performed on the liver sections of mice with the anti-F4/80 antibody (Abcam, ab100790). The images were acquired with a microscope (Carl Zeiss AG, Jena, Germany).

### Determination of Inflammatory and Oxidative Parameters

Frozen colonic and hepatic tissues as well as serum were homogenized with radioimmunoprecipitation assay lysis buffer (Solarbio, Beijing, China) to extract total proteins. Total protein level was quantified with a bicinchoninic acid protein assay kit (Solarbio, Beijing, China). The concentrations of IL-6, IL-1β, and IL-10 were measured by ELISA kits (R&D Systems, Minneapolis, MN, USA). The levels of total superoxide dismutases (T-SOD), catalase (CAT), glutathione peroxidase (GSH-px), and malondialdehyde (MDA) were quantified using commercial kits (Nanjing Jiancheng Bioengineering Institute, Nanjing, China) according to the manufacturer’s directions.

### Biochemical Analysis

The contents of myeloperoxidase (MPO), aspartate aminotransferase (AST), and alanine aminotransferase (ALT) were assayed using commercial kits (Nanjing Jiancheng Bioengineering Institute, Nanjing, China) according to the manufacturer’s directions.

### RNA Sequencing

Total RNA of colonic and hepatic tissues was isolated using TRIzol™ Reagent (Invitrogen, CA, USA) and purified using a PureLink™ RNA Mini Kit (Invitrogen, CA, USA) according to the manufacturer’s directions. RNA quality was evaluated by electrophoresis using an Agilent 2100 Bioanalyzer (Agilent Technologies, CA, USA). Samples with RNA integrity number >9.4 and with 260/280 nm absorbance ratio from 1.9 to 2.1 were used for the construction of the library products for RNA sequencing. The library products were prepared using the TrugSeq™ RNA Sample Prep kit (Illumina, CA, USA) according to the manufacturer’s directions. Then, sequencing of the library products was performed in Illumina HiseqTM 2500 (Illumina, CA, USA), and the quality was individually assessed using FastQC. Based on the referential genome of *Mus musculus* (version GRCm38.p6), mapped reads were acquired, and then differentially expressed genes were identified using the DEGseq2 package. Heat maps were generated using the “pheat-map” packages of R software (version 4.1.2; https://www.r-project.org/). Scatter plots were derived using RSEM (version 1.3.3). The data were analyzed through the free online platform of Majorbio Cloud Platform (www.majorbio.com). Gene Ontology (GO) enrichment was performed using Goatools (version 0.6.5) based on the GO database (version 2019.7.1; http://www.geneontology.org/).

### Sample Collection, DNA Extraction, and 16s rRNA Sequencing

Total DNA was extracted from fecal samples using QIAamp^®^ Fast DNA Stool Mini Kits (Qiagen Ltd., Germany) according to the manufacturer’s instructions. Then, the V3–V4 region of the 16s rRNA gene was amplified using universal primers 338F (5′-ACTCCTACGGGAGGCAGCAG-3′) and 806R (5′-GGACTACHVGGGTWTCTAAT-3′). The PCR cycling programs were 95°C for 3 min, 29 cycles of 95°C for 30 s, 55°C for 30 s, and 72°C for 45 s, with a final extension at 72°C for 10 min. Agarose gel electrophoresis was performed to verify the quality and size of the amplicons. After purification and quantification, the normalized PCR products were pooled in equal volumes and sequenced on an Illumina MiSeq 2 × 300-bp paired-end sequencer. Negative controls for DNA extraction, amplification, and mock community (Zymo, Irvine, CA, USA) were included in each MiSeq run for quality control.

### Microbiota Data Analysis

Raw sequences were analyzed using the QIIME platform (version 1.9.1). Multiplex single-end sequencing reads (>50,000 per sample) were imported into the QIIME1.9 platform. The initial reads were quality-filtered, denoised, and assembled. Chimeric sequences were removed using data2. The subsequent clean reads were clustered as operational taxonomic units (OTUs) using Uparse (version 7.0.1090) and annotated with the SILVA 16S rRNA gene database (version 132) using MOTHUR program (version 1.30.2). Alpha-diversity was calculated based on the profiles of OTU. Beta-diversity was estimated by calculating the unweighted and weighted UniFrac distances (Bray–Curtis distance and 999 permutations) and then visualized with principal coordinate analysis (PCoA). ANOSIM was performed to compare the similarity of bacterial communities among groups using the “vegan” package of R (version 4.1.2). To identify the differential bacteria among groups, the relative abundances of the phylum and genus levels were analyzed by Kruskal–Wallis *H*-test and adjusted by false discovery rate. Bar plots and heat maps were done using the “ggplot2” packages of R software (version 4.1.2; https://www.r-project.org/), respectively. All the relevant scripts for analysis are shown in **Additional File 1**. Differentially abundant genera were identified using linear discriminant analysis (LDA) effect size (LEfSe) analysis (http://huttenhower.sph.harvard.edu/galaxy/root?tool_id=lefse_upload) (30). Only bacterial taxa reaching the LDA threshold of 2.0 and with average relative abundances greater than 0.01% are shown.

### RNA Extraction and Real-Time Quantitative PCR

The colonic tissues were subjected to total RNA isolation, cDNA synthesis, and RT- qPCR as we have previously described (31). The qPCR reactions were performed on LightCycler^®^ 96 Real-Time PCR System (Roche Molecular Systems, MA, Switzerland) as follows: 95°C for 1 min, followed by 40 cycles of 95°C for 10 s and another 10 s at the respective annealing temperature for each gene. Melting curve analysis was performed to verify the specificity of the PCR reactions. *Reg3b* and *Reg3g* expressions were assayed by real-time PCR using SYBR Green Supermix (Takara, Tokyo, Japan) with specific primers (forward: TCCCAGGCTTATGGCTCCTA, reverse: GCAGGCCAGTTCTGCATCA; forward: TTCCTGTCCTCCATGATCAAAA, reverse: CATCCACCTCTGTTGGGTTCA) (13). *β-actin* was used as a control (forward: AGGTGACAGCATTGCTTCTG, reverse: GCTGCC TCAACACCTCAAC) (32). Gene expression was normalized to the housekeeping gene *β-actin*.

### Statistical Analysis

All data are presented as means ± SEM and analyzed using GraphPad Prism (version 9.0, GraphPad Software, San Diego, CA, USA). Data among groups were compared using one-way ANOVA, followed by Tukey’s multiple-comparison tests. Adjusted *P* ≤0.05 was considered statistically significant. Heat maps (correlation between parameters from the colon and liver) were created using the “pheat-map” packages of R software (version 4.1.2). All the relevant scripts for analysis are shown in**Additional File 1**.

## Results

### MFGM Attenuated DSS-Induced Acute Colitis in Mice

Firstly, the alleviated effects of prophylactic MFGM in DSS-induced colitis were assessed. The body weight of mice was significantly increased in the MFGM + DSS group compared with the DSS group (**Figure 1B**), but the body weight was not impacted by MFGM in healthy mice (mice without DSS) (**Figure 1B**). Decreased DAI score was shown in the MFGM + DSS group compared with the DSS group (**Figure 1C**). The colonic length was also shortened by DSS treatment, while it was increased in the MFGM-treated group (**Figures 1D, E**). H&E staining revealed that the histological score of colons was increased in the DSS group but was significantly decreased in mice with prophylactic MFGM (**Figures 1F, G**).

DSS treatment significantly increased the colonic levels of IL-1β and IL-6 but decreased the colonic level of IL-10, MPO, and MDA, while these were reversed by MFGM (**Figures 1H–L**).

### MFGM Altered the Colonic Gene Expression Profiles Associated With Epithelial Cell Proliferation and Response to Protozoan in DSS-Treated Mice

**Figure 2:**
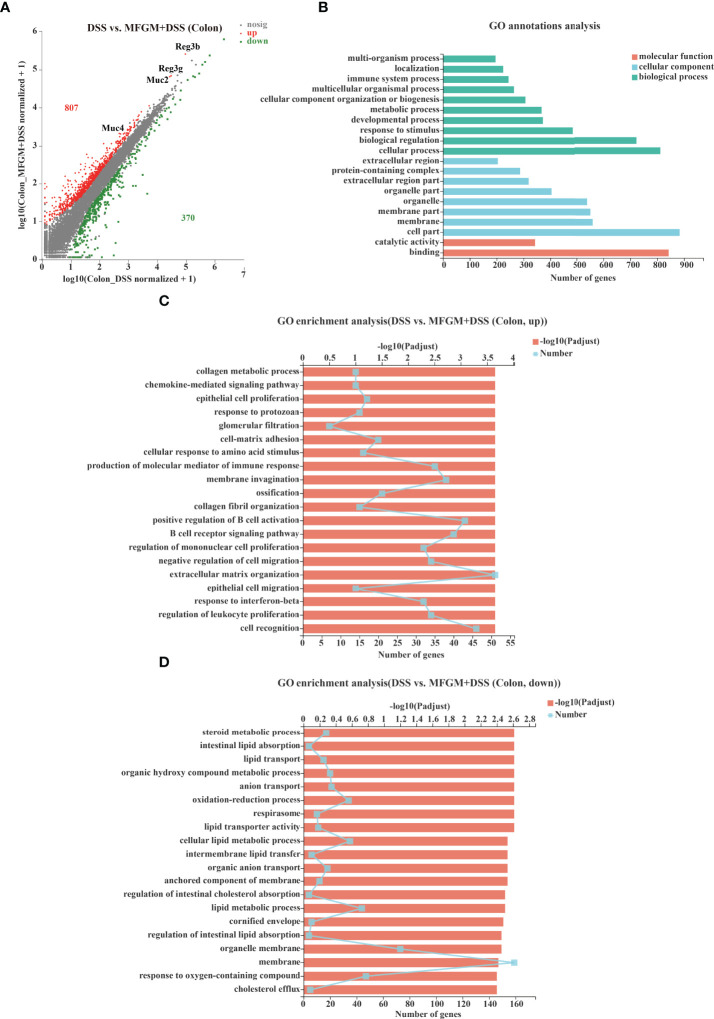
Milk fat globule membrane (MFGM) impacted the colonic gene expression profiles in dextran sulfate sodium (DSS)-treated mice. **(A)** Scatter diagram of the differentially expressed genes in the comparison. The red point denotes an upregulated gene in the MFGM + DSS group, while the green point denotes a downregulated gene in the MFGM + DSS group compared with the DSS group. The gray point denotes an insignificantly expressed gene in the comparison. **(B)** Gene Ontology (GO) annotation analysis of the differentially expressed genes in the comparison of the colon. GO enrichment of the upregulated genes **(C)** and the downregulated genes **(D)** in the comparison of the colon.

To explore how MFGM protects from colonic damage, the gene expression profiles for colonic tissues were quantified by RNA-seq analysis. The gene expression profiles of colonic tissue were significantly influenced by prophylactic MFGM (**Supplementary Figure S2**). In total, 807 genes were significantly upregulated, but 370 genes were significantly downregulated in the MFGM + DSS group (**Figure 2A**). Genes including *Ighv1-72*, *Ighv1-53*, *Ighv1-82*, *Ighv1-61*, *Igkv4-51*, *Igkv2-116*, *MUC2*, *MUC4*, *Reg3b*, and *Reg3g* were twice upregulated in the MFGM + DSS group compared with the DSS group (**Figure 2A**).

Furthermore, the functions of the altered genes were annotated by GO pathway analysis. The enriched pathways of the comparison were mainly enriched in the parts of biological process and cellular component, consisting of cellular process, cell part, and binding shown by GO analysis (**Figure 2B**). Moreover, the differential genes from the comparison were also respectively determined by GO enrichment analysis. The upregulated pathways of the comparison were consisting of such as chemokine-mediated signaling pathway, epithelial cell proliferation, response to protozoan, and cell matrix adhesion in the MFGM + DSS group compared with the DSS group (**Figure 2C**). On the contrary, some metabolic pathways such as steroid metabolic process, intestinal lipid absorption, and lipid transport were downregulated in colitis mice with prophylactic MFGM (**Figure 2D**).

### MFGM Modulated the Gut Microbiota Community of Mice

**Figure 3:**
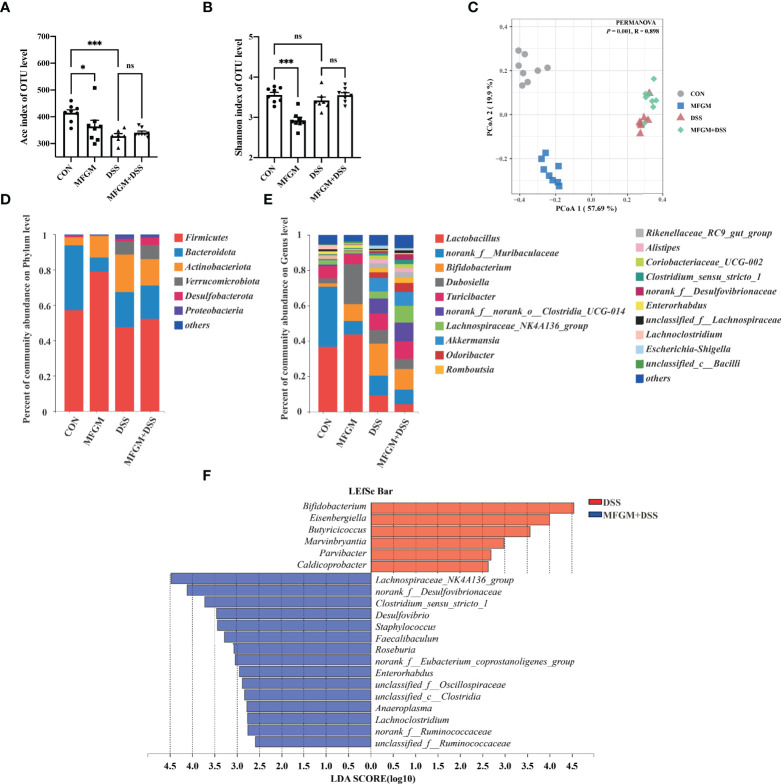
Milk fat globule membrane (MFGM) regulated the composition and structure of intestinal microbiota. α-Diversity upon oral therapy represented by the Ace index **(A)** and Shannon index **(B)**. **(C)** Principal coordinate analysis plots upon dextran sulfate sodium (DSS) treatment. The relative abundance of fecal bacterial phyla **(D)** and genera **(E)** presented in 99.5% of the community upon DSS treatment. **(F)** Analysis of differences in the microbial taxa shown by linear discriminant analysis coupled with effect size measurements upon DSS treatment. Data were presented as means ± SEM (*n* = 6–8 per group). Statistical significance was determined using one-way ANOVA, followed by Tukey’s test. ns, no significant, *P ≤ 0.05, ***P ≤ 0.001.

To further explore how prophylactic MFGM impacts the response to protozoan, the community of gut microbiota was explored by 16s rRNA V3–V4 sequencing. Interestingly, prophylactic MFGM significantly decreased the community richness and community diversity of the fecal microbiota in both mice with and without DSS, shown by the ACE estimator and Shannon diversity index (**Figures 3A, B**). The PCoA revealed the respective clustering of gut microbiota for the four groups (*R* = 0.898, *P* = 0.001, **Figure 3C**) and a significantly separated clustering of microbiota between groups with MFGM and without MFGM in healthy mice (*R* = 0.858, *P* = 0.002) as well as between the DSS group and the MFGM + DSS group (*R* = 0.634, *P* = 0.002). At the phylum level, all fecal samples shared a similar community structure. The phyla in all groups were mainly dominated by *Firmicutes* and *Bacteroidetes*, representing more than 80% of the relative abundance in healthy mice but more than 60% of it in DSS-treated mice (**Figure 3D**). At the genus level, *Lactobacillus*, *norank_f:Muribaculaceae*, *Bifidobacterium*, and *Dubosiella* were the dominant genera in all groups (**Figure 3E**). Furthermore, differential bacteria at the genus level were identified by LEfSe. The LEfSe analysis revealed that six genera were enriched in the DSS group, while another 15 genera, such as *Faccalibacumum* and *Roseburia*, were enriched in the MFGM + DSS group (**Figure 3F**). Nine genera, including *Bifidobacterium* and *Dubosiella*, were significantly impacted by MFGM compared to the normal controls (**Supplementary Figure S3**).

### MFGM Attenuated DSS-Induced Liver Injury in Mice

**Figure 4:**
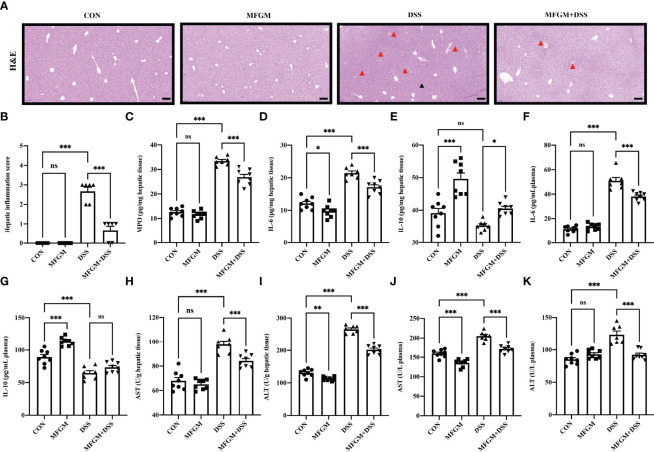
Milk fat globule membrane alleviated hepatic injury secondary to dextran sulfate sodium-induced experimental colitis. **(A)** H&E-stained liver sections and **(B)** hepatic inflammation scores of livers (*n* = 6 per group). Scale bars represent 100 μm. The infiltration of immunocytes was marked by black triangles, and local bleeding was marked by red triangles. Concentrations of MPO **(C)**, IL-6 **(D)**, and IL-10 **(E)** in the liver from each group. Levels of IL-6 **(F)** and IL-10 **(G)** in the plasma. Concentrations of AST **(H)** and ALT **(I)** in the liver (*n* = 6–8 per group). Concentrations of AST **(J)**, and ALT **(K)** in the plasma (*n* = 6–8 per group). Data are presented as means ± SEM. Statistical significance was determined using one-way ANOVA, followed by Tukey’s test. ns, no significant, *P < 0.05, **P < 0.01, ***P < 0.001.

In fact, liver injury was always along with acute colitis (10). Notably, the gut–liver axis consisting of the gut microbiota, intestinal mucosal barrier, and hepatic immunity might play a critical role in secondary liver injury (14, 15, 17). Therefore, the attenuated effect of MFGM in colitis-associated liver injury was further explored. H&E staining revealed that DSS induced histological damage in the liver, as shown by the infiltration of immunocytes, and even local bleeding, which were alleviated in mice with MFGM (**Figures 4A, B**). The hepatic MPO level was significantly enhanced by DSS compared to normal controls, while it was significantly attenuated in the MFGM + DSS group (**Figure 4C**). The hepatic levels of MPO, IL-6, and IL-10 were also increased in the DSS-treated mice, while they were decreased in the MFGM+DSS group (**Figures 4D, E**). Increased plasma levels of IL-6 and IL-10 were shown in mice with prophylactic MFGM compared to the DSS group (**Figures 4F, G**). Furthermore, the concentrations of AST and ALT in the plasma and liver were obviously evaluated by DSS treatment, while they were reduced in mice with prophylactic MFGM compared with the DSS group (**Figures 4H–K**).

### MFGM Altered the Hepatic Gene Expression Profiles Related to Glutathione Transferase Activity in DSS-Treated Mice

**Figure 5:**
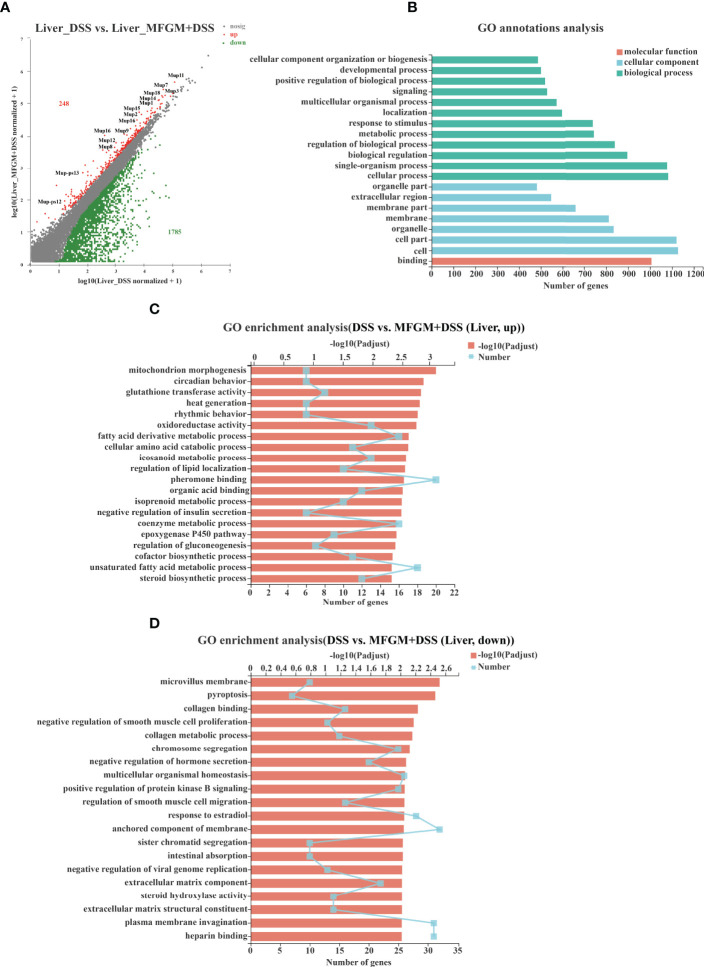
Milk fat globule membrane (MFGM) impacted the gene expression profile of liver in dextran sulfate sodium (DSS)-treated mice. **(A)** Scatter diagram of the differentially expressed genes in the comparison. The red point denotes an upregulated gene in the MFGM + DSS group, while the green point denotes a downregulated gene in the MFGM + DSS group compared with the DSS group. The gray point denotes an insignificantly expressed gene in the comparison. **(B)** Gene Ontology (GO) annotation analysis of the differentially expressed genes in the comparison of the liver. GO enrichment of the upregulated genes **(C)** and the downregulated genes **(D)** in the comparison of the liver.

To further assess the impact of prophylactic MFGM on hepatic function, hepatic gene expression profiles of DSS-treated mice were also quantified by RNA-seq analysis (**Supplementary Figure S1**). In total, 248 genes were upregulated, while 1,785 genes were downregulated in the liver of the MFGM + DSS group compared with the DSS group (**Figure 5A**). Of note is that major urine proteins (MUPs), such as *MUPs1–3* and *MUPs7–9*, were twice upregulated in the MFGM+DSS group than in the DSS group, as shown with a scatter plot (**Figure 5A**). The GO annotation analysis revealed that impacted genes were mostly enriched in cellular process, cell, and binding (**Figure 5B**). The upregulated pathways were mainly consisting of such as mitochondrion morphogenesis, circadian behavior, glutathione transferase activity, and oxidoreductase activity (**Figure 5C**). Otherwise, pathways such as microvillus membrane, pyroptosis, collagen binding, collagen metabolic process, and sister chromatid segregation were downregulated in the MFGM + DSS group compared to the DSS group (**Figure 5D**).

### MFGM Maintained the Intestinal Mucosal Barrier and Protected the DSS-Treated Mice From Oxidative Stress of the Liver

**Figure 6:**
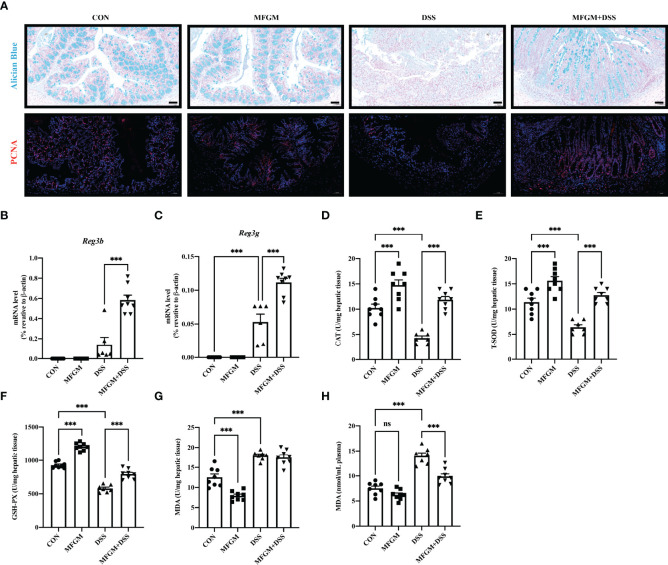
Milk fat globule membrane (MFGM) maintained the colonic mucus barrier and regulated the oxidative stress of the liver. **(A)** Representative images of Alcian blue-stained inner mucus layer and proliferating cell nuclear antigen-stained colonic epithelia of colonic sections. Scale bars represent 50 μm. mRNA expression of Reg3b **(B)** and Reg3g **(C)** in the colon. Concentrations of CAT **(D)**, T-SOD **(E)**, GSH-PX **(F)**, and MDA **(G)** in the hepatic tissues. **(H)** Level of MDA in the plasma (*n* = 6–8 per group). Data are presented as means ± SEM (*n* = 8 per group). Statistical significance was determined using one-way ANOVA, followed by Tukey’s test. ns, no significant, ***P ≤ 0.001.

**Figure 7:**
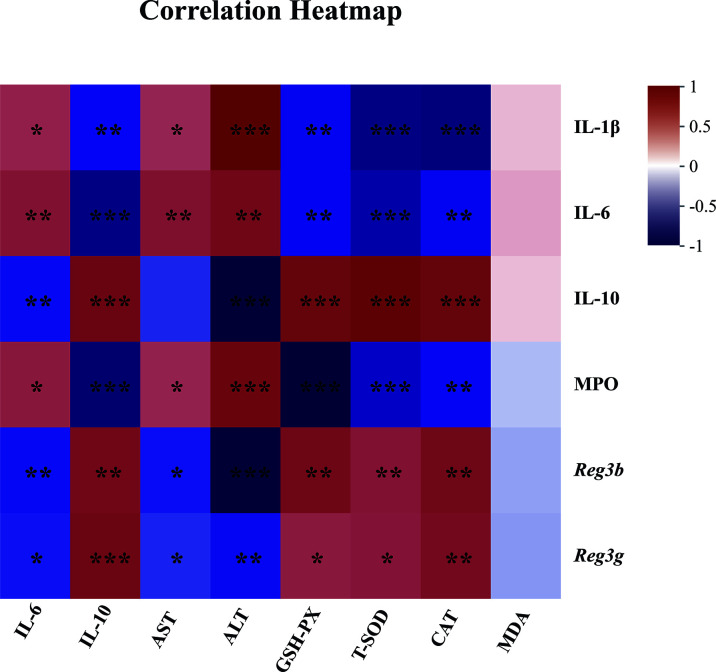
Spearman correlation between anti-inflammatory or anti-oxidative parameters of colonic tissues (vertical axis) and hepatic tissues (horizontal axis) in dextran sulfate sodium-treated mice. The red color denotes a positive correlation, while the blue color denotes a negative correlation. The intensity of the color is proportional to the strength of the Spearman correlation. *P ≤ 0.05, **P ≤ 0.01, ***P ≤ 0.001.

Since “epithelial cell proliferation” and “response to protozoan” was enriched in the colonic RNA-seq data, these two pathways were further determined in the colonic samples. Epithelial cell proliferation was accessed by PCNA staining. The results revealed that the expression of positive PCNA was significantly increased in the MFGM + DSS group compared with the DSS group (**Figure 6A**). Moreover, the decreased mucus layer of the colon upon DSS treatment was revealed by Alcian blue staining, while it was increased in the MFGM + DSS group (**Figure 6A**). To further demonstrate it, the colonic RNA expression levels of *Reg3b* and *Reg3g* were quantified by qPCR. Similarly, both significantly upregulated levels of *Reg3b* and *Reg3g* were revealed in the MFGM + DSS group compared with mice treated with DSS (**Figures 6B, C**). All these data suggested that prophylactic MFGM improved the colonic epithelial barrier. Moreover, since glutathione transferase activity was enriched in the MFGM + DSS group shown by GO analysis, the hepatic levels of CAT, T-SOD, GSH-PX, and MDA were explored. Significantly increased hepatic levels of CAT, T-SOD, and GSH-PX while decreased hepatic and plasma levels of MDA were shown in the MFGM + DSS group (**Figures 6D–H**). The correlation between the parameters of colonic tissues and hepatic tissues in DSS-treated mice was also analyzed. The results revealed that the colonic concentrations of IL-1β, IL-6, and MPO positively correlated with the hepatic levels of IL-6, AST, and ALT and negatively correlated with the hepatic levels of IL-10, GSH-PX, T-SOD, and CAT (**Figure 7**; **Additional Files 2**, **3**). Moreover, the colonic levels of IL-10, *Reg3b*, and *Reg3g* showed a negative correlation with the hepatic levels of IL-6, AST, and ALT, but with a positive correlation with the hepatic levels of IL-10, GSH-PX, T-SOD, and CAT (**Figure 7**; **Additional Files 2**, **3**).

## Discussion

The beneficial effects of MFGM in modulating metabolic diseases associated with dysbiosis (33) as well as normalization of the intestinal homeostasis have been shown in recent studies (22, 34). However, the effects and mechanisms of MFGM in dysbiosis associated with gut inflammation and secondary hepatic injury are still poorly known. Here we revealed that MFGM can alleviate acute colitis and secondary liver injury in mice. Moreover, the elevated levels of intestinal Reg3 lectins as well as the enhanced mucosal barrier function and the balanced bacterial community might contribute to the alleviation in colitis and hepatic injury.

We found that MFGM can alleviate DSS-induced acute colitis, as shown by the decreased body weight loss and DAI score as well as less shortening of the colonic length and histological damage of the colon. Pre-supplementation of MFGM also improved the inflammatory status of the colon caused by DSS. Similarly, MFGM could decrease the levels of IL-1β and IL-6 induced by lipopolysaccharide *in vivo* and *in vitro* (25). Our previous study has demonstrated that mixed supplementation of milk bioactive components, consisting of MFGM, fructo-oligosaccharides, and galacto-oligosaccharides, alleviated colitis by shifting the phenotype of lamina propria macrophages and thereby reducing the production of pro-inflammatory cytokines (28). However, the specific effect of MFGM on colitis is still unknown. MPO, a lysosomal protein found in neutrophils, serves as a viable biomarker for assessing the status of the disease (3, 35). The level of MPO in the active IBD patients was significantly increased compared with that in healthy controls or inactive IBD patients. We provide the evidence that prophylactic MFGM protected the mice from DSS-induced colonic damage and inflammation.

The colonic gene expression profile revealed that the pathways associated with barrier functions, such as epithelial cell proliferation and response to protozoan, were enriched in MFGM-administrated mice after DSS treatment. Epithelial cell proliferation is reported to be associated with preventing inflammation in the gut (36). MFGM improved the intestinal physical barrier by impacting the epithelial cell proliferation. On the contrary, MFGM has been reported to affect the anti-proliferative activity of colon cancer cells (37). Meanwhile, response to protozoan can be impacted by the gut microbiome, a stable mucus barrier, enrichment of Reg3 lectins, and host immune response (38, 39). The colonic mucus layer is a dynamic and chemically complex barrier composed largely of secreted MUC2. MUC2 was mainly expressed in small intestines, colons, and tracheobronchial tissues, while MUC4 was expressed in virtually all epithelia (40). Increased expressions of *MUC2*, *MUC4*, *Reg3b*, and *Reg3g* revealed that MFGM promoted a chemical barrier *via* the enrichment of the secretion of mucins and antimicrobial peptides. Of note is that some other genes (*Ighvs* and *Igkvs*) were significantly upregulated, indicating that the production of immune globulins might further protect the mucus barrier from bacteria. Consistent with it, our previous study has found that MFGM promoted the expression of *MUC2* and tight junction protein (*ZO-1*, *occuludin*, and *claudin-1*) in intrauterine growth restriction mice (25).

Previous studies have revealed that both CD and UC gut microbiomes exhibit general decreases in *Firmicutes* (41). The enrichment of *Firmicutes* indicated that MFGM led to a lower risk of the gut microbial community in inducing colitis. *Roseburia* and *Faecalibacterium* genera are decreased in CD patients (42). This may explain the enrichment of *Firmicutes* and *Dubosiella* in MFGM-administrated mice after DSS treatment. Moreover, as the abundance of *Dubosiella* and *Bifidobacterium* was highly enriched in MFGM-treated mice than those in normal healthy controls, these findings suggested that MFGM contributes to improve the microbial barrier.

Recently, it has been reported that many IBD patients also suffer from a secondary liver injury (43). Consistent with previous studies, the hepatic histological damage was observed in DSS-induced murine colitis model (43, 44). AST and ALT always seemed like sensitive indicators to access the hepatic damage (45, 46). Here our results revealed the increased plasma and hepatic levels of AST and ALT in acute colitis mice. The hepatic inflammatory injury was significantly alleviated by prophylactic MFGM. To explore how MFGM attenuated the secondary hepatic damage, the gene expression profiles of liver in DSS-treated mice were analyzed. The RNA-seq analysis of the liver indicated that prophylactic MFGM improved the normal chemical communication, enhanced the metabolism of glucose and lipid, and promoted the anti-oxidative capacity in DSS-treated mice. In agreement with it, previous studies revealed that human milk components containing MFG exert anti-oxidative capacities by acting as radical scavengers and modulating enzyme activity and enzyme expression (47, 48). The decreased level of MDA and the increased level of SOD were also previously reported in the intestine of rat and IEC-6 enterocytes upon MFGM (49). That suggested a strongly anti-oxidative status in hepatic tissues, and a decreased level of oxidative stress in the liver was revealed in DSS-treated mice with prophylactic MFGM.

As noted in previous studies, hepatitis was often established along with acute colitis or dysbiosis of gut microbiota (10, 16). The liver–gut axis has highlighted the close interactions among the intestinal mucosal barrier, gut microbiota, and hepatic immunity (14, 16, 17). Accordingly, we assumed that the alleviation of MFGM in colitis and hepatic injury was associated with the protection for mucus layer. Therefore, the correlation between the inflammatory cytokines (IL-1β, IL-6, and IL-10) and the parameters related to tissue injury in the colon and liver revealed that hepatic injury was associated with DSS-induced colitis, which was in agreement with the gut–liver axis demonstrated by previous studies (14–16). Moreover, the correlation between Reg3 lectin mRNA levels and the parameters linked with oxidative stress in the colonic and hepatic tissues indicated that the MFGM-induced alleviation in hepatic damage was associated with the protection of the mucus barrier.

We concluded that MFGM supplementation protected the mice from DSS-induced colitis and hepatic injury by increasing the gene levels of intestinal Reg3 lectins as well as improving the mucosal barrier and bacterial community of the colon and further inhibiting oxidative stress of the liver. It is reasonable to expand our study into other hepatitis models such as steatohepatitis and alcoholic hepatitis and provide potential therapy for IBD and secondary hepatic injury.

## Data Availability Statement

The datasets supporting the conclusions of this article are available in the NCBI Sequence Read Archive (SRA) repository under accession number PRJNA765454 and PRJNA766403 (available on October 10, 2021).

## Ethics Statement

The animal study was reviewed and approved by the Institutional Animal Care and Use Committee of the China Agricultural University.

## Author Contributions

DH and JW designed the experiments. ZW, XL, SH, and TL conducted the experiments. ZW, XL, JP, XZ, and BZ collected the samples and performed the analysis of samples. ZW, XL, SH, XZ, TL, JZ, and LC analyzed the data. ZW wrote the manuscript. All authors contributed to the article and approved the submitted version.

## Funding

This work was supported by the National Natural Science Foundation of China (31902170, 31630074, and 32172750), the Beijing Municipal Natural Science Foundation (S170001), the China Agriculture Research System of MOF and MARA (CARS-35), and the 111 Project (B16044).

## Conflict of Interest

Authors JZ and LC are employed by company Beijing Sanyuan Foods Co. Ltd.

The remaining authors declare that the research was conducted in the absence of any commercial or financial relationships that could be construed as a potential conflict of interest.

## Publisher’s Note

All claims expressed in this article are solely those of the authors and do not necessarily represent those of their affiliated organizations, or those of the publisher, the editors and the reviewers. Any product that may be evaluated in this article, or claim that may be made by its manufacturer, is not guaranteed or endorsed by the publisher.

## Supplementary Material

Supplementary Figure 1 Heat map summary of the differentially expressed genes in the comparison of the liver.

Supplementary Figure 2 Heat map summary of the differentially expressed genes in the comparison of the colon.

Supplementary Figure 3 Analysis of differences in the microbial taxa shown by linear discriminant analysis coupled with effect size measurements between the two groups without dextran sulfate sodium treatment.

Additional File 1 Full account of the statistical analysis performed in R software (version 4.1.2).

Additional File 2 Correlation coefficient values of the correlation analysis.

Additional File 3
*P*-values of the correlation analysis.

## Abbreviations

IBD, inflammatory bowel disease; MFGM, milk fat globule membrane; DSS, dextran sulfate sodium; DAI, disease activity index; MPO, myeloperoxidase; IL, interleukin; AST, aspartate aminotransferase; ALT, alanine aminotransferase; CAT, catalase; T-SOD, total superoxide dismutases; GSH-PX, glutathione peroxidase; MDA, malondialdehyde; Reg3, regenerating islet derived-3; SPF, specific pathogen-free; PBS, phosphate-buffered saline; H&E, hematoxylin and eosin; PCNA, proliferating cell nuclear antigen; BCA, bicinchoninic acid; RINs, RNA integrity numbers; GO, Gene Ontology; OTUs, operational taxonomic units; PCoA, principal coordinate analysis; FDR, false discovery rate; LDA, linear discriminant analysis; LEfSe, linear discriminant analysis effect size; MUPs, major urine proteins; LPS, lipopolysaccharide
