# Supplementary figures and images for: Milk Fat Globule Membrane Attenuates Acute Colitis and Secondary Liver Injury by Improving the Mucus Barrier and Regulating the Gut Microbiota
Source: Front Immunol. 2022 Jun 21;13:865273. doi: 10.3389/fimmu.2022.865273 (PMC9253277; doi:10.3389/fimmu.2022.865273)

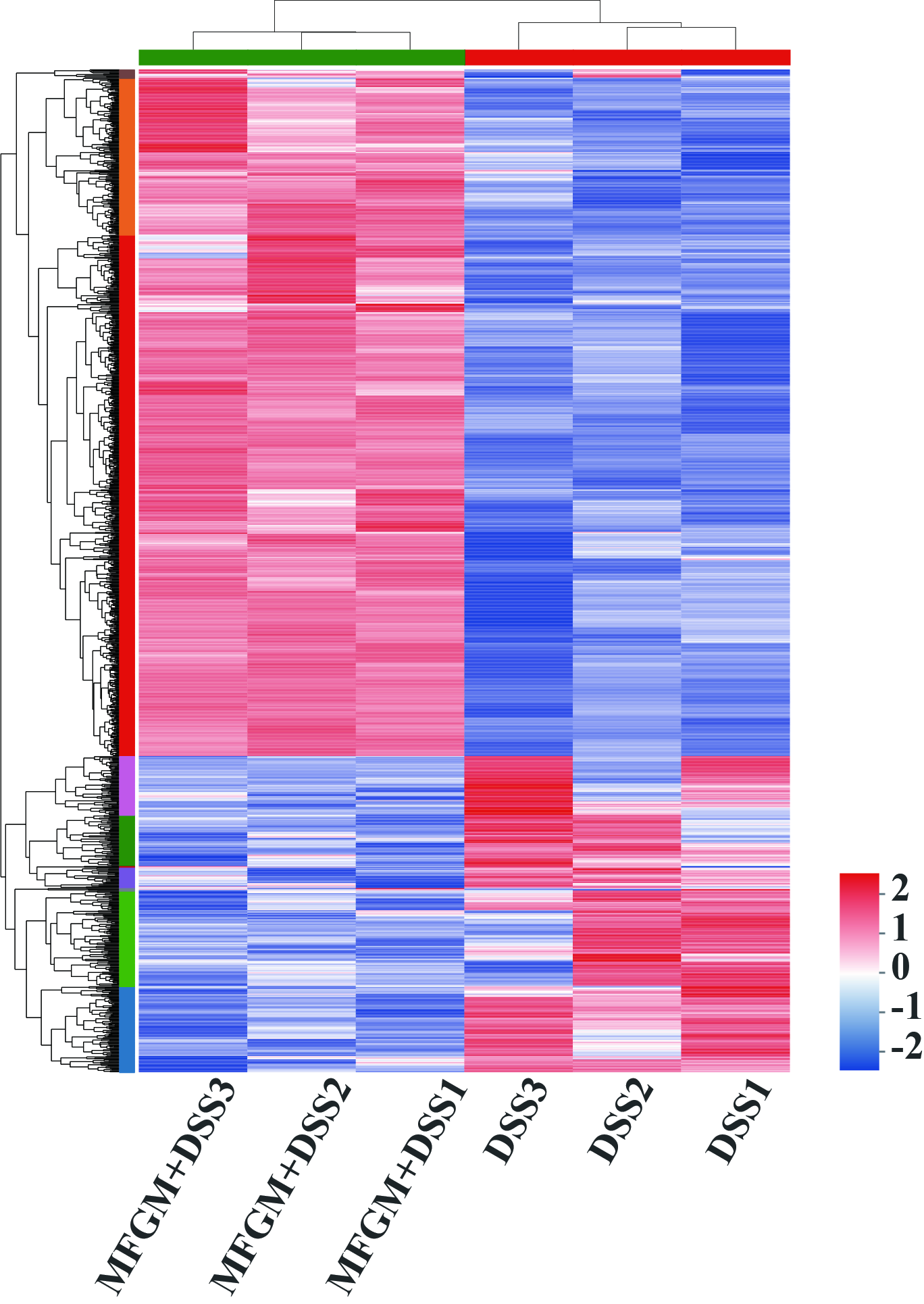

Supplement: Supplementary Figure 1 — Heat map summary of the differentially expressed genes in the comparison of the liver. [file Image_1.tif]

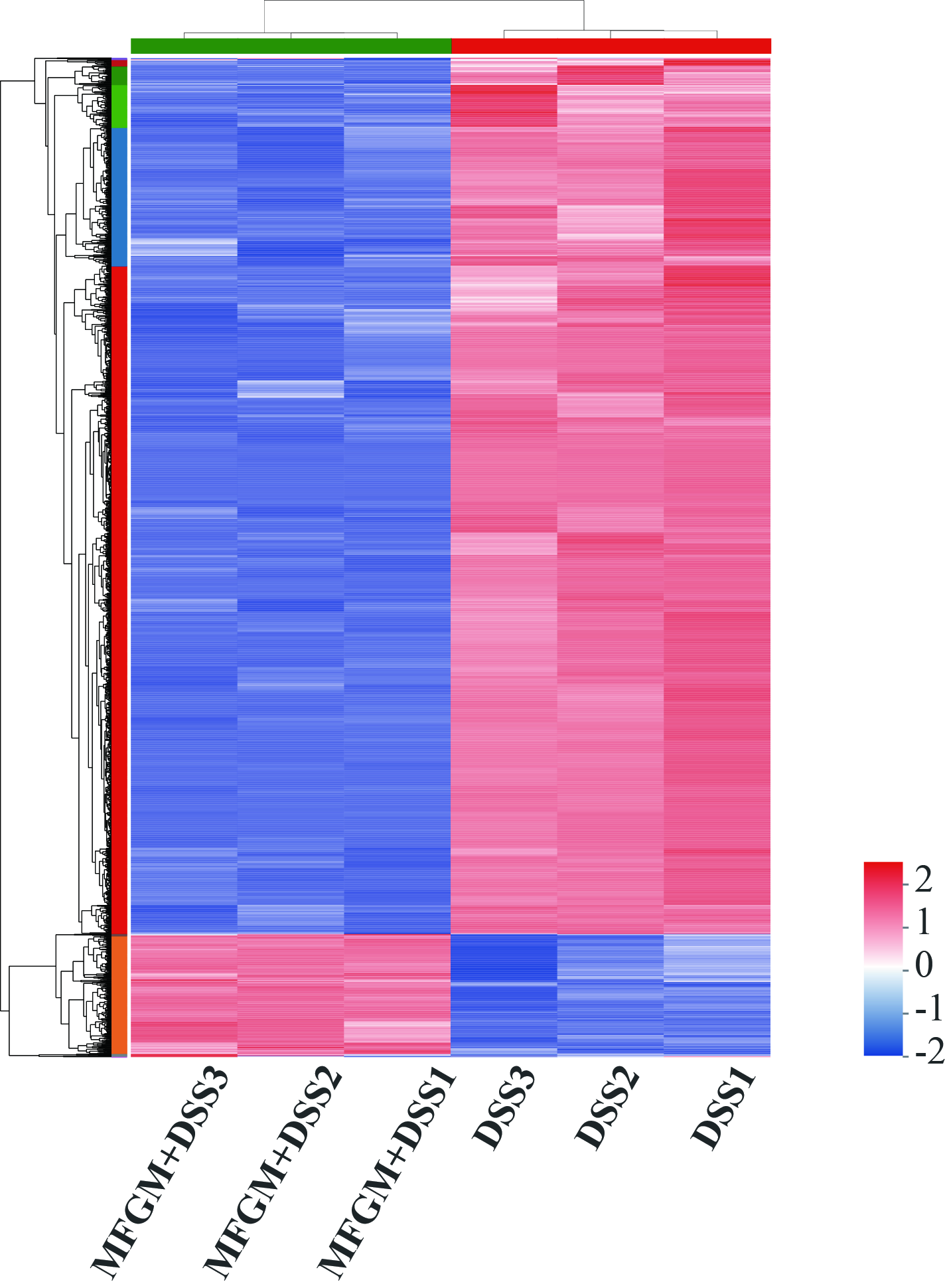

Supplement: Supplementary Figure 3 — Analysis of differences in the microbial taxa shown by linear discriminant analysis coupled with effect size measurements between the two groups without dextran sulfate sodium treatment. [file Image_3.tif]
