# Supplementary material for: Milk Fat Globule Membrane Attenuates Acute Colitis and Secondary Liver Injury by Improving the Mucus Barrier and Regulating the Gut Microbiota
Source: Front Immunol. 2022 Jun 21;13:865273. doi: 10.3389/fimmu.2022.865273 (PMC9253277; doi:10.3389/fimmu.2022.865273)
Supplement: Additional File 1 — Full account of the statistical analysis performed in R software (version 4.1.2). [file Table_1.docx]

This document contains all the scripts used for the analyses in the data analysis section.

**Results:**

How was the gut microbiota community in colitis mice or normal mice impacted by MFGM?

Firstly, the code for software loading using R software (version 4.1.2).

The following workspace contains:

- feature-table.txt: The OTU levels of all samples.
- feature-table2.txt: The OTU levels of the CON and MFGM group.
- feature-table3.txt: The OTU levels of the DSS and MFGM+DSS group.
- taxonomy.txt: The OTUs and their corresponding Kindom, Phylum, Class, Order, Family, Genus, and Species.
- metadata.txt: Samples ID and its corresponding group of all samples.
- metadata2.txt: Samples ID and its corresponding group of the CON and MFGM group.
- metadata3.txt: Samples ID and its corresponding group of the CON and MFGM group.

| setwd("/Users/verawu/desktop/microbiota_analysis/")  rm(list = ls())  set.seed(123)  library(ggplot2)  library(microbiome)  library(MicrobiotaProcess)  library(treeio)  library(ggsci)  library(vegan)  library(extrafont)  library(tidyr)  library(reshape2)  library(Hmisc)  library(conflicted)  conflict_prefer("tax_table", "phyloseq")  conflict_prefer("transform", "microbiome")  conflict_scout()  feature <- read.delim("feature-table.txt", row.names = 1, header = T)  taxonomy <- read.delim("taxonomy.txt", row.names = 1, header = T)  metadata <- read.delim("metadata.txt", row.names = 1, header = T)  metadata$treatment <- factor(metadata$treatment, levels = c("CON", "MFGM", "DSS", "MFGM_DSS"))  feature <- floor(feature+0.5)  feature <- as.matrix(feature) %>%  otu_table(taxa_are_rows = T)  taxonomy <- as.matrix(taxonomy) %>%  tax_table()  MFGM_1 <- phyloseq(feature, taxonomy, sample_data(metadata))  summarize_phyloseq(MFGM_1)  ntaxa(MFGM_1)  alpha_diversity <- get_alphaindex(MFGM_1)  alpha_diversity_output <- as.data.frame(alpha_diversity) |
| --- |

The Alpha diversity was analyzed.

| alpha_diversity <- get_alphaindex(MFGM_1)  alpha_diversity_output <- as.data.frame(alpha_diversity) |
| --- |

PERMANOVA analysis was performed. The PCoA plots were generated based on Bray-Curtis distances and permutations with the Adonis function available.

| MFGM_1rel <- transform(MFGM_1, transform = "compositional")  MFGM_abundance <- abundances(MFGM_1rel)  MFGM_metadata <- meta(MFGM_1rel)  permanova <- adonis(t(MFGM_abundance) ~ treatment,  data = MFGM_metadata, permutations = 999, method = "bray")  print(as.data.frame(permanova$aov.tab)["treatment", "Pr(>F)"])  feature <- read.delim("feature-table.txt", row.names = 1, header = T)  taxonomy <- read.delim("taxonomy.txt", row.names = 1, header = T)  metadata <- read.delim("metadata.txt", header = T)  feature <- as.data.frame(t(feature))  metadata$treatment <- as.factor(metadata$treatment)  distance_bray <- vegdist(feature, method = 'bray')  pcoa_bray <- cmdscale(distance_bray, k = 18, eig = TRUE)  pcoa_eig <- (pcoa_bray$eig)[1:2] / sum(pcoa_bray$eig)  sample_site <- data.frame({pcoa_bray$point})[1:2]  sample_site$SampleID <- rownames(sample_site)  names(sample_site)[1:2] <- c('PCoA1', 'PCoA2')  sample_site <- merge(sample_site, metadata, by = 'SampleID', all.x = TRUE)  sample_site$treatment <- factor(sample_site$treatment, levels = c('CON', 'MFGM', 'DSS', 'MFGM_DSS'))  ggplot(sample_site) +  geom_point(aes(PCoA1, PCoA2, color = treatment, shape = treatment), size = 4, alpha = 0.8) +  scale_color_manual(values=c("#999999", "#2874C5", "#CC6666", "#66CC99")) +  scale_shape_manual(values = c(16, 15, 17, 18)) +  theme_bw() +  labs(x = paste('PCoA 1 (', round(100 * pcoa_eig[1], 2), '%)'),  y = paste('PCoA 2 (', round(100 * pcoa_eig[2], 2), '%)')) +  annotate("text", x = 0.25, y = 0.4, label = "PERMANOVA \n P = 0.001, R^2^ = 0.807", size = 3)  ggsave("pcoa.pdf", width = 5, height = 4) |
| --- |

For the two groups treated without DSS:

| feature <- read.delim("feature-table2.txt", row.names = 1, header = T)  taxonomy <- read.delim("taxonomy.txt", row.names = 1, header = T)  metadata <- read.delim("metadata2.txt", row.names = 1, header = T)  metadata$treatment <- factor(metadata$treatment, levels = c("CON", "MFGM"))  feature <- as.matrix(feature) %>%  otu_table(taxa_are_rows = T)  taxonomy <- as.matrix(taxonomy) %>%  tax_table()  MFGM_2 <- phyloseq(feature, taxonomy, sample_data(metadata))  summarize_phyloseq(MFGM_2)  ntaxa(MFGM_2)  MFGM_2 <- phyloseq(feature, taxonomy, sample_data(metadata))  summarize_phyloseq(MFGM_2)  MFGM_2rel <- transform(MFGM_2, transform = "compositional")  MFGM_abundance <- abundances(MFGM_2rel)  MFGM_metadata <- meta(MFGM_2rel)  permanova2 <- adonis(t(MFGM_abundance) ~ treatment,  data = MFGM_metadata, permutations = 999, method = "bray")  print(as.data.frame(permanova$aov.tab)["treatment", "Pr(>F)"]) |
| --- |

For the two groups treated with DSS:

| feature <- read.delim("feature-table3.txt", row.names = 1, header = T)  taxonomy <- read.delim("taxonomy.txt", row.names = 1, header = T)  metadata <- read.delim("metadata3.txt", row.names = 1, header = T)  metadata$treatment <- factor(metadata$treatment, levels = c("DSS", "MFGM_DSS"))  feature <- as.matrix(feature) %>%  otu_table(taxa_are_rows = T)  taxonomy <- as.matrix(taxonomy) %>%  tax_table()  MFGM_3 <- phyloseq(feature, taxonomy, sample_data(metadata))  summarize_phyloseq(MFGM_3)  ntaxa(MFGM_3)  MFGM_3rel <- transform(MFGM_3, transform = "compositional")  MFGM_abundance <- abundances(MFGM_3rel)  MFGM_metadata <- meta(MFGM_3rel)  Permanova3 <- adonis(t(MFGM_abundance) ~ treatment,  data = MFGM_metadata, permutations = 999, method = "bray")  print(as.data.frame(permanova$aov.tab)["treatment", "Pr(>F)"]) |
| --- |

And, the composition of microbial community was performed.

The relative abundance on phylum level:

| MFGM_1rel <- transform(MFGM_1, transform = "compositional")  MFGM_1_phylum <- get_taxadf(obj=MFGM_1rel, taxlevel=2, detection = 0.005)  ggbartax(obj=MFGM_1_phylum) +  xlab(NULL) + ylab("Relative abundance (%)")  ggsave("MFGM_phylum.pdf ", width = 15, height = 10) |
| --- |

The relative abundance on genus level:

| MFGM_1_genus<- get_taxadf(obj= MFGM_1rel, taxlevel=7)  MFGM_1_genus <- get_taxadf(obj=MFGM_1rel, taxlevel=6, detection = 0.005)  ggbartax(obj=MFGM_1_genus, average_by = "treatment") + xlab(NULL) + ylab("relative abundance (%)")  ggsave("MFGM_genus.pdf", width = 15, height = 10 |
| --- |

Moreover, the correlation heatmaps between paraments from colon and liver were performed.

The following workspace contains:

- sample(liver).csv: anti-inflammatory or anti-oxidative parameters of liver.
- sample(colon).csv: anti-inflammatory or anti-oxidative parameters of colon.

| setwd("/Users/verawu/desktop/microbiota_analysis/")  rm(list = ls())  set.seed(123)  library(psych)  library(pheatmap)  library(reshape2)  library(RColorBrewer)  MFGMcolon<-read.delim('sample(colon).csv', row.names = 1, sep = ',', stringsAsFactors = FALSE, check.names = FALSE)  MFGMliver<-read.delim('sample(liver).csv', row.names = 1, sep = ',', stringsAsFactors = FALSE, check.names = FALSE)  Correlation <- corr.test(MFGMcolon, MFGMliver, method="spearman", adjust="none")  MFGMp <- Correlation$p  MFGMr <- Correlation$r  col <- colorRampPalette(c("navy","white", "firebrick3"))(100)  pheatmap(MFGMr, fontsize_number=14,fontsize = 14,cluster_rows = F,  display_numbers = matrix(ifelse(MFGMp <= 0.001 ,"***",  ifelse(MFGMp <= 0.01, "**",  ifelse(MFGMp <= 0.05 ,"*"," "))), nrow(MFGMp)),  cluster_cols = FALSE,fontface = "bold",number_color = "black",border_color = 'grey30',  color = col,angle_col = "45",cellwidth = 24, cellheight = 20) |
| --- |
